# High Mortality in Adults With Community-Acquired Pneumonia Admitted to Hospitals in South Africa Before and During the COVID-19 Pandemic, Stratified by HIV Serostatus and Viral Load: Data From a Prospective Cohort Study (2019–2021)

**DOI:** 10.1093/ofid/ofag594

**Published:** 2026-09-21

**Authors:** Nadia Sabet, Firdaus Nabeemeeah, Kennedy Otwombe, Khuthadzo Hlongwane, Lebohang M Mlambo, Tumelo Moloantoa, Michelle Wong, Phetho Mangena, Pattamukkil Abraham, Floris Swanepoel, Anne von Gottberg, Cheryl Cohen, Mignon du Plessis, Jo Southern, Mark A Fletcher, Eileen M Dunne, Bradford D Gessner, Bha Ndungane-Tlakula, Minja Milovanovic, Ebrahim Variava, Neil A Martinson

**Affiliations:** Perinatal HIV Research Unit, University of the Witwatersrand, Johannesburg, South Africa; Perinatal HIV Research Unit, University of the Witwatersrand, Johannesburg, South Africa; Perinatal HIV Research Unit, University of the Witwatersrand, Johannesburg, South Africa; School of Public Health, Faculty of Health Sciences, University of the Witwatersrand, Johannesburg, South Africa; Perinatal HIV Research Unit, University of the Witwatersrand, Johannesburg, South Africa; School of Public Health, Faculty of Health Sciences, University of the Witwatersrand, Johannesburg, South Africa; Perinatal HIV Research Unit, University of the Witwatersrand, Johannesburg, South Africa; Perinatal HIV Research Unit, University of the Witwatersrand, Johannesburg, South Africa; Department of Internal Medicine, Faculty of Health Sciences, University of the Witwatersrand, Johannesburg, South Africa; Division of Pulmonology, Department of Internal Medicine, Chris Hani Baragwanath Academic Hospital, Johannesburg, South Africa; Department of Internal Medicine, University of Limpopo, Polokwane, South Africa; Department of Internal Medicine, Polokwane Hospital, Polokwane, South Africa; Perinatal HIV Research Unit, University of the Witwatersrand, Johannesburg, South Africa; Perinatal HIV Research Unit, University of the Witwatersrand, Johannesburg, South Africa; Centre for Respiratory Diseases and Meningitis, National Institute for Communicable Diseases of the National Health Laboratory Service, Johannesburg, South Africa; School of Public Health, Faculty of Health Sciences, University of the Witwatersrand, Johannesburg, South Africa; Centre for Respiratory Diseases and Meningitis, National Institute for Communicable Diseases of the National Health Laboratory Service, Johannesburg, South Africa; Centre for Respiratory Diseases and Meningitis, National Institute for Communicable Diseases of the National Health Laboratory Service, Johannesburg, South Africa; Medical Evidence Development Vaccines, Chief Medical Affairs Office, Pfizer Inc, Collegeville, Pennsylvania, USA; Pfizer Vaccines Research & Development, Medical Affairs, International Emerging Markets, Paris, France; Medical Evidence Development Vaccines, Chief Medical Affairs Office, Pfizer Inc, Collegeville, Pennsylvania, USA; Medical Evidence Development Vaccines, Chief Medical Affairs Office, Pfizer Inc, Collegeville, Pennsylvania, USA; EpiVac Consulting, Anchorage, Alaska, USA; Pfizer, Johannesburg, South Africa; Perinatal HIV Research Unit, University of the Witwatersrand, Johannesburg, South Africa; African Potential Collaborative, Faculty of Health Sciences, University of the Witwatersrand, Johannesburg, South Africa; Perinatal HIV Research Unit, University of the Witwatersrand, Johannesburg, South Africa; Department of Internal Medicine, Faculty of Health Sciences, University of the Witwatersrand, Johannesburg, South Africa; Division of Pulmonology, Department of Internal Medicine, Chris Hani Baragwanath Academic Hospital, Johannesburg, South Africa; North West Province Department of Health, Klerksdorp/Tshepong Hospital Complex, Klerksdorp, South Africa; Perinatal HIV Research Unit, University of the Witwatersrand, Johannesburg, South Africa; Center for TB Research, Johns Hopkins University, Baltimore, Maryland, USA

**Keywords:** community-acquired pneumonia, COVID-19, HIV, mortality, tuberculosis

## Abstract

**Background:**

Community-acquired pneumonia (CAP) is a leading cause of death in adults, but recent data in people with HIV (PWH) are lacking. We studied South African adults hospitalized with CAP to describe mortality and risk factors.

**Methods:**

We prospectively enrolled consenting adults aged ≥18 years hospitalized with CAP, as defined by symptoms, signs, and chest x-ray, from March 2019 to October 2021 in 3 South African hospitals. Blood, sputum, nasopharyngeal/oropharyngeal swabs, and urine were tested for HIV, tuberculosis, SARS-CoV-2, influenza, respiratory syncytial virus, and *Streptococcus pneumoniae*. Participants were followed for 1 year. Causes of death were defined following record review. Timing and risk factors for death were evaluated by Kaplan-Meier and Cox analyses.

**Results:**

Of 2000 adults (median age, 46 years), 1079 (54.0%) were PWH. Among 994 with HIV viral load results, 289 (29.1%) were virally suppressed, and among 1556 tested for SARS-CoV-2, 700 (45.0%) were positive. There were 569 (28.5%) deaths, and the majority died early: 47.7% during hospitalization and 10.6% ≤30 days postdischarge. Leading underlying causes of death were COVID-19 (23.0%), tuberculosis (16.9%), and advanced HIV (13.2%). Age ≥45 years (hazard ratio, 2.7; 95% CI, 1.5–4.6), active tuberculosis (1.6, 1.2–2.0), unsuppressed HIV viral load (1.4, 1.0–1.9), anemia (1.3, 1.0–1.6), underweight (1.3, 1.0–1.7), and low admission oxygen saturation (<94% on room air; 1.3, 1.0–1.6) were associated with mortality. Mortality was higher in PWH (18.2 vs 11.7 per 100 person-years) but similar between PWH who were virally suppressed and unsuppressed (21.3 vs 24.7 per 100 person-years).

**Conclusions:**

In-hospital and postdischarge CAP mortality remains high, particularly among PWH, regardless of virologic status, warranting urgent investigation of mitigation strategies.

## Teaser

Mortality among South African adults hospitalized with community-acquired pneumonia, including COVID-19, is high (∼30%). Mortality remains high in people with HIV despite widespread antiretroviral therapy, strengthened tuberculosis control, and indirect benefits from routine childhood pneumococcal vaccination programs.

Community-acquired pneumonia (CAP) is the leading infectious disease cause of morbidity and mortality worldwide, with the greatest burden borne by low- and middle-income countries where limited diagnostic capacity, high prevalence of comorbidities and coinfections, and late access to care confound estimations [1]. *Streptococcus pneumoniae* predominates as the cause of pneumonia in adults. In South Africa, prevention and reduction strategies include empiric antibiotic treatment and indirect effects from childhood pneumococcal vaccination, as there is no implemented adult vaccination program and hence extremely limited data on the latter [2, 3].

Risk of CAP and death related to CAP is increased in people with HIV-1 infection (PWH), particularly in those with advanced disease, in whom opportunistic pathogens such as *Mycobacterium tuberculosis* and *Pneumocystis jirovecii* remain common [4, 5]. Scale-up of HIV test-and-treat strategies has accelerated progress to viral suppression targets, and in South Africa, the majority of an estimated 7 million people receiving antiretroviral therapy (ART) are suppressed [6]. However, hospitalizations due to CAP are associated with high inpatient and postdischarge mortality [7], contributing to longevity differences when people without HIV (PWoH) are compared with those with HIV [8, 9].

Contemporaneous data from settings with high HIV prevalence that include ART and viral suppression status are limited [10]. We therefore assessed early and late mortality in a cohort of patients with CAP admitted to hospital between May 2019 and October 2021 (before and during the COVID-19 pandemic) to describe risk factors for mortality and inform potential preventive and therapeutic strategies.

## METHODS

### Study Design and Participants

Our prospective cohort (HospCAP), nested within the study “The Potentially Preventable Burden of Community-Acquired Pneumonia in South African Adults in the Era of Widespread PCV13 Immunisation and Antiretroviral Therapy Roll-out,” is described in detail elsewhere [11]. Briefly, adults (aged ≥18 years) enrolled within the first 48 hours of hospitalization for a presumptive diagnosis of CAP—based on the standardized clinical case definition of severe acute respiratory infection [12]—were screened between May 2019 and October 2021 at 3 academic hospitals in South Africa. The hospitals differed by regional HIV prevalence and childhood vaccination uptake per the Expanded Program on Immunization, respectively indicated in the parentheses that follow [13], and adult vaccination coverage data are not available:

- Chris Hani Baragwanath Academic Hospital in Soweto, Gauteng province (15.0%, 81.4%)
- Klerksdorp-Tshepong Hospital Complex in Klerksdorp, North-West province (16.5%, 75.2%)
- Polokwane-Mankweng Hospital Complex in Polokwane, Limpopo province (11.9%, 70.5%) [6, 13]

From the HospCAP cohort, a convenience subsample was recruited of all eligible individuals who could be approached, provide consent, and have samples taken during working hours. We excluded those who were considered terminal, who were previously hospitalized in the last 30 days, who lacked a chest x-ray (CXR), or who were concurrently enrolled in clinical trials for pneumonia or pneumonia vaccination.

Participant recruitment halted briefly (March–June 2020) due to COVID-19 lockdowns, and an increase to the original sample size was implemented based on preliminary high-mortality data from early recruitment. The first confirmed case of SARS-CoV-2 pneumonia in South Africa (5 March 2020) marked the onset of the “COVID era” in this analysis [11].

### Procedures

Demographic data were obtained from participant or next-of-kin interviews, clinical data were abstracted from medical records, and laboratory results preceding and during participant admissions were recorded. Routinely collected CXRs taken within 48 hours of hospital admission were used to confirm pulmonary pathology and eligibility, except in pneumocystis pneumonia or COVID-19 where clinical signs of pneumonia taken with laboratory confirmation were accepted in lieu of CXR findings because of the possibility of limited radiologic findings despite severe clinical presentation [14, 15]. HIV status was defined for all participants by clinical records of a positive HIV rapid test and/or plasma HIV viral load (HIVVL) result, laboratory-based enzyme-linked immunosorbent assay on blood, or, where not available, an HIV rapid and confirmatory test performed by the study team. In PWH, baseline CD4 cell count and HIVVL were obtained at enrollment. Viral suppression was defined as HIVVL ≤50 copies/mL. Investigations were performed for influenza virus A and B, respiratory syncytial virus (RSV), *S pneumoniae*, SARS-CoV-2 (upon its emergence, often routinely at admission and again by study team on recruitment), and *M tuberculosis*. *P jirovecii* infection was investigated where clinically suspected as part of routine care. Smoking status was ascertained through self-report and semiquantitative urinary cotinine assays [11].

### Follow-Up and Outcomes

Participants were monitored daily during admission to ascertain clinical course, new diagnoses, discharge status, or death. During follow-up, participants were contacted by telephone every week or 2 weeks (in summer months) and had in-person clinic visits every 4 months. To ascertain vital status, participants who did not answer telephone calls or missed follow-up visits were traced through next-of-kin contacts or home visits [11]. In the event of a death, 2 medically qualified study investigators (F. N., N. S.) independently reviewed available medical records, laboratory results (including those available postmortem), imaging data, family interviews, and death certificates. Deaths were categorized as “natural” or “unnatural” (eg, trauma or suicide) and the latter excluded in mortality analyses. For natural deaths, we documented a single underlying cause of death, without which the death would not have occurred and which most likely initiated the train of mortal events [16]. For example, if a participant with tuberculosis (TB) were being treated and responding well but developed a pulmonary embolus and died, the cause of death would be ruled “pulmonary embolus.” This was determined by consensus, adapting methods from prior work in the same population [17, 18].

Primary mortality outcomes were in hospital, ≤30 days of hospital discharge, and 31 days postdischarge up to 1 year as, due to COVID-related study disruptions, not all participants could be followed up for the full 2 years as originally planned. Death postdischarge could have occurred at home or following hospital readmission. Risk-scoring tools CURB-65 [19], ACHU [20], and NEWS2 [21] assessed pneumonia severity [22].

### Data Analysis

Categorical variables were compared with χ^2^ tests by study outcome (survived vs died) and HIV status (PWoH, PWH who were virally suppressed, PWH who were virally unsuppressed). The Kruskal-Wallis test was used to compare continuous measures. In our comparisons by HIV status, 85 of 1079 (7.9%) PWH and 12 of 334 (3.6%) PWH who died had no HIVVL available; these were included in initial descriptions of the cohort but excluded from stratified analyses. Kaplan-Meier plots and log-rank tests were used to describe and compare probability of death, stratified by HIV status, during hospitalization and ≤30 days postdischarge (early mortality) and 31 days to 1 year postdischarge (late mortality). A portion (63.0%) of the cohort was followed beyond 1 year, but we restrict cohort analyses to those with up to 1 year of follow-up. Cox proportional hazard regression was used to determine risk factors associated with time-dependent mortality. Three models were performed: during hospitalization, ≤30 days after discharge from hospital, and 31 days after discharge up to 1 year. Time was computed from hospital admission until death, relocation, loss to follow-up, or end of follow-up ≤1 year. Overall and stratified relative contribution to overall mortality was reported as percentages of the total for cause of death and as a rate per 100 person-years of follow-up stratified by sex, age group, and HIV serostatus. The univariate model included demographic information, body mass index, HIV status, TB status, and laboratory measures. Variables that were significant in the univariate model were initially included in a full multivariate model, and stepwise selection procedures were used to arrive at the final model. Statistical analysis was conducted in SAS Enterprise Guide 8.6 (SAS Institute Inc). Risk scores were excluded from multivariable analyses due to collinearity, but risk score components (eg, blood pressure or age) were included.

### Patient Consent Statement

All participants provided written informed consent. The study was approved by the University of the Witwatersrand Human Research Ethics Committee (171009) and by the study sites’ provincial and hospital research ethics committees.

## RESULTS

### Enrollment and Patient Characteristics

Of 6546 screened adults with a presumptive diagnosis of pneumonia seen in the emergency departments of hospitals from May 2019 to October 2021, 2000 eligible participants were enrolled in the cohort (Figure 1) [11].

**Figure 1.**
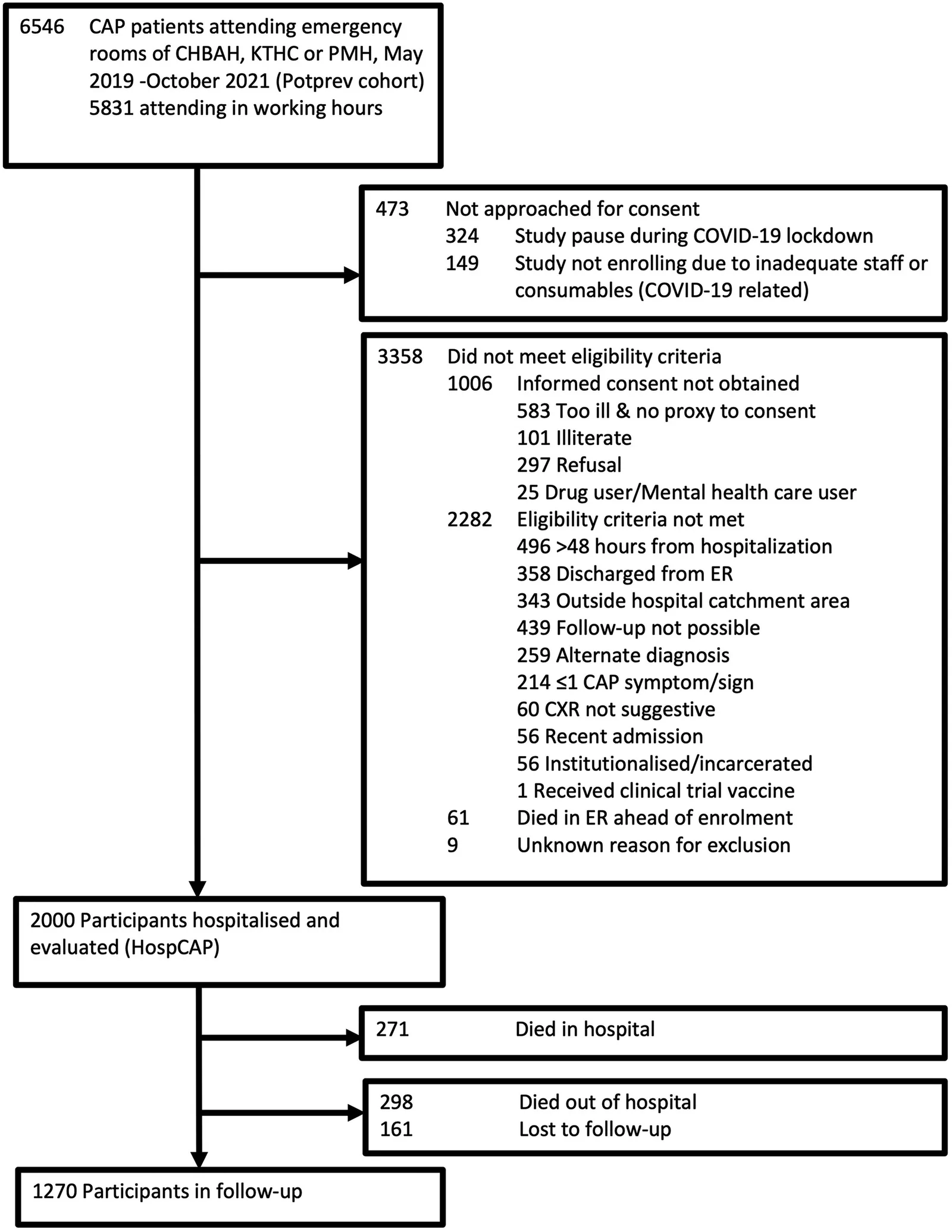
Participant flow for the HospCAP cohort, derived from the larger multisite PotPrev Study, South Africa, 2019–2021, as described by Nabeemeeah et al [11]. CAP, community-acquired pneumonia; CHBAH, Chris Hani Baragwanath Academic Hospital; CXR, chest x-ray; ER, emergency room; KTHC, Klerksdorp-Tshepong Hospital Complex; PMH, Polokwane-Mankweng Hospital.

Of enrolled participants, one-third (668/2000, 33.4%) were aged ≥55 years and half (985/2000, 49.3%) were male (Table 1). HIV prevalence overall was 54.0% (1079/2000; 95% CI, 52.0%–56.0%) with a median time of 3.0 years (IQR, 0–7) from self-reported HIV diagnosis to enrollment. PWH were younger (median, 41 years; IQR, 34–50) than PWoH (56, 41–64; *P* < .001) and more likely to be female (57.0% vs 43.0%, *P* = .005). HIVVL results were available in 994 of 1079 (92.1%) of all PWH, among whom 289 (29.1%) were virally suppressed at admission. Most participants reported at least a 7-day history of respiratory symptoms (1345/2000, 67.3%). Although 486 of 2000 (24.3%) participants reported a history of treated TB disease, not all had chronic lung disease (Table 1).

**Table 1.** Baseline Characteristics of PotPrev HospCAP Cohort at 3 Hospitals in South Africa, 2019–2021

	No. (%) or Median (IQR)		
	Overall (N = 2000)	Survived (n = 1431)	Died (n = 569)
Hospital			
CHBAH	914 (45.7)	627 (43.8)	287 (50.4)
KTHC	865 (43.3)	645 (45.1)	220 (38.7)
PMHC	221 (11.1)	159 (11.1)	62 (10.9)
Age, y (N = 2000)	46 (36–58)	45 (35–57)	50 (37–62)
Male	985 (49.3)	684 (47.8)	301 (52.9)
PWH	1079 (54.0)	745 (52.1)	334 (58.7)
Viral load suppressed^a^	289/994 (29.1)	208/672 (31.0)	81/322 (25.2)
Viral load unsuppressed	705/994 (70.9)	464/672 (69.0)	241/322 (75.8)
CD4 count^b^ (n = 1040)	109 (33–276)	133 (44–317)	61 (14–178)
Self-reported ART	653/1077 (60.6)	462/743 (62.2)	191/334 (57.2)
Comorbidities			
Cardiovascular disease	514 (25.7)	367 (25.7)	147 (569 (25.8)
Diabetes	204 (10.2)	149 (10.4)	55 (9.7)
Chronic respiratory disease	109 (5.5)	70 (4.9)	39 (6.9)
Other	53 (2.7)	42 (2.9)	11 (1.9)
Body mass index, kg/m^2^ (n = 1911)	24.2 (19.5–30.5)	25.1 (20.0–31.5)	22.2 (18.0–28.0)
Underweight	378/1911 (19.8)	229/1380 (16.6)	149/531 (28.1)
Obese	519/1911 (27.2)	417/1380 (30.2)	102/531 (19.2)
Chest x-ray abnormal	1841 (92.1)	1306 (91.3)	535 (94.0)
TB diagnosis^c^	513/1919 (26.7)	315/1370 (23.0)	198/549 (36.1))
Newly diagnosed at admission	478/513 (93.2)	292/315 (92.7)	186/198 (93.9)
SARS-CoV-2 positive	700/1556 (45.0)	540/1133 (47.7)	160/423 (37.8)
UAD positive^d^	339/1733 (19.6)	256/1256 (20.4)	83/477 (17.4)
Blood lytA positive^e^	109/1948 (5.6)	86/1394 (6.2)	23/554 (4.2)
Self-reported smoker			
Current	391 (19.6)	293 (20.5)	98 (17.2)
Previous smoker	364 (18.2)	224 (15.7)	140 (24.6)
Excessive alcohol use^f^	88/684 (12.9)	68/514 (13.2)	20/170 (11.8)
Cough duration, d (n = 1550)	7 (3–14)	6 (3–14)	7 (3–14
Hospital stay, d (N = 2000)	8 (5–12)	8 (4–11)	8 (5–14)
Outcome of admission			
Discharged	1617 (80.9)	1339 (93.6)	278 (48.9)
Died during admission	271 (13.6)	…	271 (47.6)
Other	112 (5.6)	92 (6.4)	20 (3.5)
Follow-up, d (N = 2000)	484 (113–687)	588 (446–707)	7 (0–161)
Lost to follow-up	161 (8.1)	161 (11.3)	0 (0.0)

Denominators are per column heading unless stated otherwise.

Abbreviations: ART, antiretroviral therapy; CHBAH, Chris Hani Baragwanath Academic Hospital; KTHC, Klerksdorp-Tshepong Hospital Complex; PMHC, Polokwane Mankweng Hospital Complex; PWH, people with HIV; TB, tuberculosis; UAD, urinary antigen detection.

^a^HIV viral load ≤50 copies/mL considered suppressed; HIV viral load not available for 85 of 1079 (7.9%) of PWH.

^b^CD4 not available for 39 of 1079 (3.6%) of PWH.

^c^By Xpert, mycobacteria growth indicator tube, urinary lipoarabinomannan, or clinical definition.

^d^UAD (serotype specific or BinaxNOW) of *Streptococcus pneumoniae*.

^e^For *S pneumoniae*.

^f^Defined as >2 units of alcohol per day in males or >1 unit per day in females.

Most participants (1557/2000, 77.9%) were enrolled during the COVID era (Supplementary Figure 1). Demographic characteristics between these periods showed that the COVID era cohort was older and had a higher prevalence of cardiovascular disease and diabetes, although with a lower prevalence of HIV, TB, or pneumococcal infection (Supplementary Table 1).

### Mortality in Follow-Up

Overall, 569 of 2000 (28.5%) participants died of any cause during the study: 301 of 569 (52.9%) were male (Table 2) and participants who died were older than those who survived (median, 50 years [IQR, 37–62] vs 45 [35–57]; *P* < .001). There were 564 natural deaths (23.7 deaths per 100 person-years) during the whole study. At study closeout, 5 of 569 (0.9%) participants who died from unnatural causes were excluded from further analyses. Case fatality rates for natural deaths were 264 of 2000 (13.2%) during hospitalization, 60 of 2000 (3.0%) at ≤30 days postdischarge, and 232 of 2000 (11.6%) at ≤1 year postdischarge. The mortality of hospitalized participants prior to the COVID-19 pandemic was 43 of 443 (9.7%), and during the COVID era, it was 228 of 1557 (14.6%) where SARS-CoV-2 pneumonia was the leading cause of death.

**Table 2.** Causes of Death in the PotPrev HospCAP Cohort, Stratified by Timing of Death, South Africa, 2019–2021

	Overall Mortality (n = 564)	Early Mortality (n = 330)	Late Mortality (n = 234)	*P* Value
COVID-19^*,**^	130 (23.1)	118 (35.8)	12 (5.1)	**<.001**
Tuberculosis**	97 (17.2)	49 (14.9)	48 (20.5)	.079
Advanced HIV and opportunistic infections	76 (13.5)	44 (13.3)	32 (13.7)	.907
Chronic lung disease^a^	52 (9.2)	12 (3.6)	40 (17.1)	**<.001**
Heart disease**	47 (8.3)	17 (5.2)	30 (12.8)	**.001**
Sepsis	40 (7.1)	21 (6.4)	19 (8.1)	.423
Malignancy**	39 (6.9)	26 (7.9)	13 (5.6)	.284
Bacterial pneumonia	28 (5.0)	22 (6.7)	6 (2.6)	**.027**
Kidney or liver disease	23 (4.1)	8 (2.4)	15 (6.4)	**.018**
Other	20 (3.5)	10 (3.0)	10 (4.3)	.432
Unknown natural cause^b^	12 (2.1)	3 (0.9)	9 (3.9)	**.017**

Unnatural deaths excluded. Early mortality: ≤30 days postdischarge. Late mortality: >30 days to 1 year postdischarge. Bold indicates *P* < .05.

^a^Tuberculosis bronchiectasis, chronic obstructive pulmonary disease, restrictive lung disease.

^b^A further 4 unknown causes were unclear to natural/unnatural etiology and are excluded.

^*^Significant difference people without HIV vs people with HIV who were virally suppressed.

^**^People with HIV who were virally suppressed vs unsuppressed.

Most people who died were classified as low risk on CURB-65 (457/569, 80.3%) and ACHU (272/569, 47.8%) scoring, and the median NEWS2 score of decedents was 5 (IQR, 4–7), rated as medium risk. Few participants were admitted to the intensive care unit (11/2000, 0.6%), of whom 7 of 11 (63.6%) died. Most participants survived admission, were discharged (1617/2000, 80.9%), and subsequently followed up.

#### Characteristics and Timing of Death

While almost half of deaths (271/564, 47.7%) occurred during initial hospitalization at 1.4 deaths per 100 person-days, a further 10.6% occurred ≤30 days postdischarge (early mortality) at 0.12 deaths per 100 person-days. Median time to death of those who died in hospital was 8.0 days (IQR, 4–14). From 31 days postdischarge to 1 year, 28.2% of deaths occurred (late mortality), at a rate of 0.002 deaths per 100 person-days. Furthermore, 13.4% of deaths occurred after >1 year of follow-up, at a rate of 0.0001 deaths per 100 person-days. In PWH, late mortality was 18.2 per 100 person-years vs 11.7 for PWoH, but within PWH, mortality was similar regardless of viral suppression status: 21.3 per 100 person-years in PWH who were virally suppressed vs 24.7 in PWH who were virally unsuppressed.

While mortality was consistently higher in PWH as compared with PWoH across the 3 sites, PWH who were virally suppressed died at a similar rate to PWH who were virally unsuppressed despite having a markedly higher median (IQR) CD4 count (216 [114–374] vs 39 [10–90], *P* < .001; Supplementary Table 4). The median age of PWH who were virally suppressed and died was approximately 11 years younger than PWoH (*P* < .001). PWH who were virally suppressed and died were more often SARS-CoV-2 positive with associated high–risk factor comorbidities, including hypertension, diabetes, or obesity, as compared with PWoH.

Kaplan-Meier curves for time to death (Figure 2) are shown for PWH who were virally suppressed, PWH who were virally unsuppressed, and PWoH. Probability of death for PWH who were virally suppressed approximates PWH who were virally unsuppressed postdischarge and for the first 6 months. Causes of death between PWH who were virally suppressed and unsuppressed did not differ regardless of timing of death, whether during admission (*P* < .001), ≤30 days postdischarge (*P* = .02), or 30 days to 1 year postdischarge (*P* < .001).

**Figure 2.**
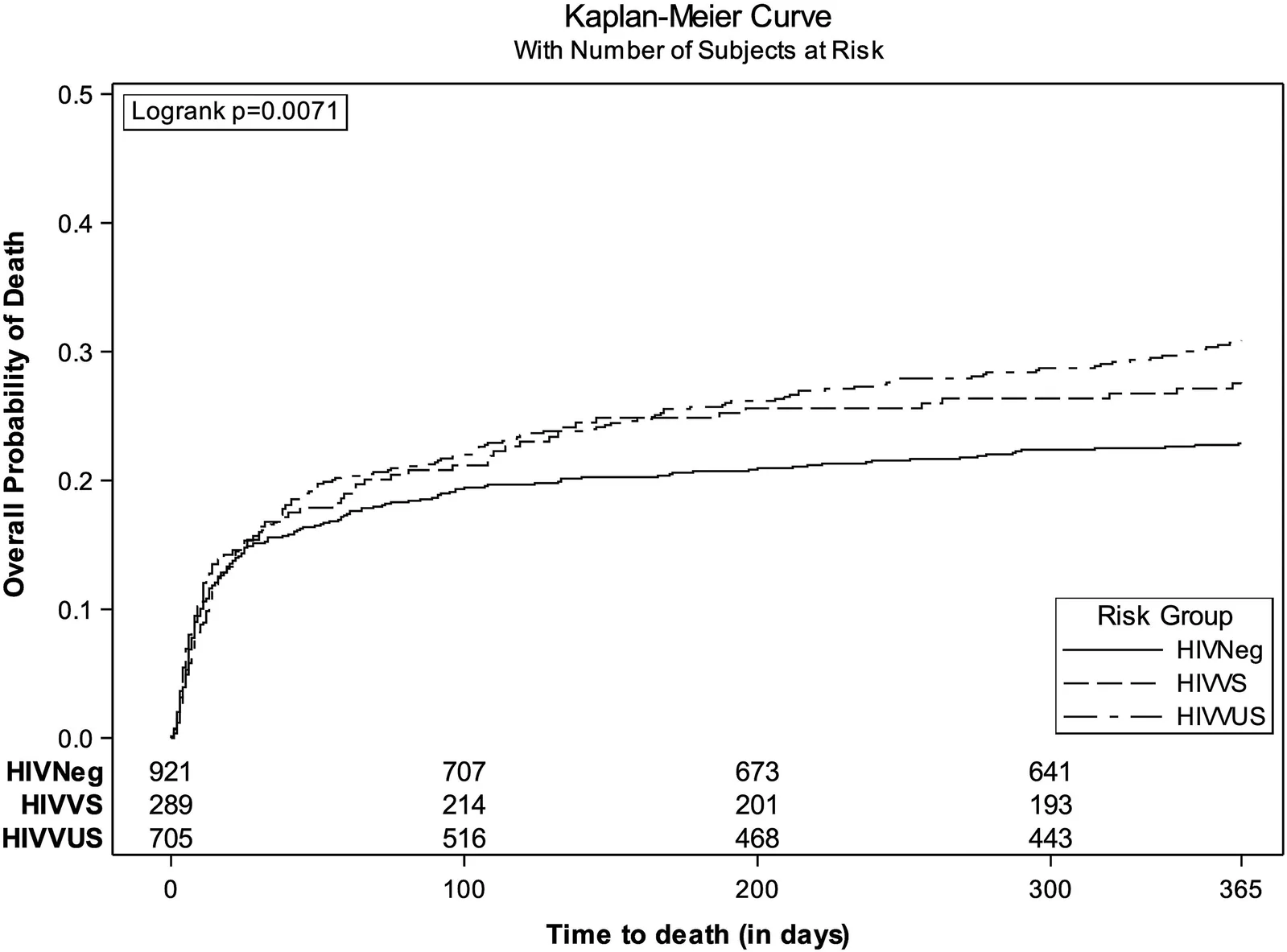
Kaplan-Meier curve in participants admitted with community-acquired pneumonia in the HospCAP cohort of the PotPrev Study to 3 hospitals in South Africa, 2019–2021, indicating early mortality and separated by HIV status and viral suppression status. HIVVS, people with HIV who were virally suppressed; HIVVUS, people with HIV who were virally unsuppressed; PWoH, people without HIV.

#### Causes of Death

While COVID-19 or advanced HIV and associated opportunistic infections (including TB, malignancy, and bacterial pneumonias) were the major underlying causes of early mortality, chronic conditions such as lung disease or heart disease occurred in those dying 31 days to 1 year postdischarge in PWoH and PWH who were virally suppressed (Table 2). PWH who were virally unsuppressed died primarily from TB, advanced HIV and other opportunistic infections, or sepsis.

### Pathogen Identification

#### SARS-CoV-2

Almost all participants recruited in the COVID-19 era were tested for SARS-CoV-2 (1556/1557, 99.9%); 700 (45.0%) tested positive (Supplementary Table 2). One-quarter (160/700, 22.9%) of participants who were SARS-CoV-2 positive died: among these, 115 (71.9%) in hospital, 12 (7.5%) ≤30 days postdischarge, and 33 (20.6%) ≤1 year postdischarge. Case fatality ratios for COVID-19 were higher in PWH, but this was not statistically significant (Supplementary Table 3). Participants with COVID-19 were older (*P* < .001), more likely to be male (*P* = .001), and more likely to have comorbidities (*P* < .001), primarily hypertension and diabetes, as compared with patients without COVID-19.

#### Tuberculosis

Overall, 1919 of 2000 (95.9%) participants had ≥1 TB specimen tested: sputum on a laboratory polymerase chain reaction assay, bedside urinary lipoarabinomannan test, or both. Of those tested, 513 (26.7%) were diagnosed with TB disease, with 478 (93.2%) newly diagnosed with TB (Table 1). Diagnosis of TB was confirmed in 258 of 513 (50.3%) with Xpert MTB/RIF Ultra and/or mycobacterial culture confirmation of *M tuberculosis*, 72 of 513 (14.0%) by urinary lipoarabinomannan alone, and 183 of 513 (36.7%) based on clinical findings and radiographic findings.

Among participants diagnosed with TB, 198 of 513 (38.6%) died. Timing of death was unavailable for 1 participant, but 84 of 197 (42.6%) died in hospital, 18 of 197 (9.1%) ≤30 days postdischarge, and 95 of 197 (48.2%) ≤1 year. Among those diagnosed by a clinician with supporting CXR without laboratory confirmation, 65 of 183 (35.5%) died despite starting anti-TB treatment.

Among those tested for TB, PWH were more likely to be *Mtb* positive (396/1071, 37.0%) than PWoH (117/848, 13.8%; *P* < .001). Case fatality ratios in patients with TB were higher in PWH than PWoH (165/296 [41.7%; 95% CI, 36.8%–46.5%] vs 33/107 [28.2%, 20.0–36.4]; Supplementary Table 3), but no significant difference was found in case fatality ratios between PWH who were virally suppressed and unsuppressed.

#### S pneumoniae

Overall, 1732 of 2000 (86.6%) participants had urine tested for *S pneumoniae* with BinaxNOW and 1716 of 2000 (85.8%) with serotype-specific urinary antigen detection (ssUAD). Of those tested by both, 339 of 1733 (19.6%) were positive on ≥1 assay: 268 of 1716 (15.6%) by ssUAD and 202 of 1732 (11.7%) by BinaxNOW. Of participants who tested positive by either assay, 83 of 339 (22.4%) died.

Blood lytA polymerase chain reaction was performed in 1948 of 2000 (97.4%) participants, of whom 109 of 1948 (5.6%) were positive and 23 of 109 (21.1%) died. PWH were 2-fold more likely to test positive for *S pneumoniae* on any pneumococcal assay than PWoH (261/1074 [24.3%] vs 110/913 [12.1%], *P* < .001). Case fatality ratios for pneumococcal pneumonia (based on a positive result by ssUAD or BinaxNOW) or invasive pneumococcal disease (based on a positive result by blood lytA) were higher in PWoH than PWH, as well as higher in PWH who were virally suppressed than unsuppressed, although neither difference reached statistical significance (Supplementary Table 3). The most frequently identified serotypes in pneumococcal-positive cases among participants who died, as detected by ssUAD, were serotypes 3 (12/73, 16.4%), 11A (8/73, 11.0%), 7F (5/73, 6.9%), and 19A (5/73, 6.9%) as compared with those who survived, in whom serotypes 3 (34/241, 14.1%), 8 (29/241, 12.0%), 9N (12/241, 5.0%), and 19A (12/241, 5.0%) were most frequently detected.

#### Other Pathogens

Very few cases of RSV (24/2953, 0.8%) or influenza A or B (42/2953, 1.4%) were detected. Most participants with a definitive result (840/2000, 42.0%) tested positive for only 1 of the 3 pathogens; coinfection occurred in a minority (83/2000, 9.2%).

#### Risk Factors for Death

In multivariable analyses, participants admitted to Chris Hani Baragwanath Academic Hospital (hazard ratio [HR], 1.4; 95% CI, 1.1–1.7; *P* = .006) had higher mortality as compared with those from Klerksdorp-Tshepong Hospital Complex (Table 3). Increased mortality was associated with the following: older age (45–54 years; HR, 1.8; 95% CI, 1.0–3.2; *P* = .048) and ≥55 years (HR, 2.7; 95% CI, 1.5–4.6; *P* < .001), being underweight (HR, 1.3; 95% CI, 1.0–1.7; *P* = .045), virally unsuppressed HIV (HR, 1.4; 95% CI, 1.0–1.9; *P* = .009), TB disease (HR, 1.6; 95% CI, 1.2–2.0; *P* < .001), anemia (HR, 1.3; 95% CI, 1.0–1.6; *P* = .028), and oxygen saturation at hospitalization of <94% on room air (HR, 1.3; 95% CI, 1.0–1.6; *P* = .031). As compared with PWH who were virally suppressed, PWoH had a 30% lower risk of death (HR, 0.7; 95% CI, .6–.9; *P* < .001).

**Table 3.** Risk Factors Associated With Mortality up to 1 Year in the PotPrev HospCAP Cohort at 3 Hospitals in South Africa, 2019–2021

	Univariable		Multivariable	
	HR (95% CI)	*P* Value	HR (95% CI)	*P* Value
Hospital				
KTHC	1 [Reference]		1 [Reference]	
CHBAH	1.4 (1.1–1.7)	**.001**	1.4 (1.1–1.7)	**.006**
PMH	1.3 (.9–1.7)	.124	1.4 (.98–1.9)	.064
Age range, y				
18–24	1 [Reference]		1 [Reference]	
25–34	1.3 (.7–2.2)	.369	1.2 (.7–2.2)	.524
35–44	1.3 (.8–2.2)	.294	1.2 (.7–2.1)	.535
45–54	1.5 (.9–2.6)	.107	1.8 (1.0–3.2)	**.048**
≥55	2.1 (1.2–3.4)	**.005**	2.7 (1.5–4.6)	**<.001**
Sex at birth				
Female	1 [Reference]		1 [Reference]	
Male	1.2 (1.0–1.4)	.050		
Body mass index				
Normal (18.5–24.9)	1 [Reference]		1 [Reference]	
Underweight (<18.5)	1.4 (1.1–1.7)	**.009**	1.3 (1.0–1.7)	**.045**
Overweight (25.0–29.9)	0.9 (.7–1.1)	.332	0.9 (.7–1.2)	.599
Obese (>30.0)	0.7 (.6–.9)	**.013**	0.9 (.7–1.2)	.457
HIV status				
Seronegative	1 [Reference]		1 [Reference]	
Virally suppressed	1.2 (.9–1.6)	.134	1.2 (.9–1.6)	.226
Virally unsuppressed	1.4 (1.1–1.7)	**.002**	1.4 (1.0–1.9)	**.009**
Confirmed tuberculosis	1.6 (1.4–2.0)	**<.001**	1.6 (1.2–2.0)	**<.001**
Tobacco smoking				
Never smoked	1 [Reference]		1 [Reference]	
Previous	1.3 (1.1–1.7)	**.008**	1.2 (.9–1.5)	.222
Current	0.9 (.7–1.1)	.281	0.9 (.7–1.2)	.531
No alcohol use	1.3 (1.0–1.5)	**.020**		
Anemia^a^	1.6 (1.3–1.9)	**<.001**	1.3 (1.0–1.6)	**.028**
Renal dysfunction^b^	1.5 (1.2–1.8)	**.001**	1.2 (.98–1.5)	.072
Random glucose^c^				
Normal	1 [Reference]		1 [Reference]	
Low	1.1 (.6–2.1)	.839		
High	0.8 (.6–1.1)	.204		
Oxygen saturation <94% on room air at admission	1.2 (.98–1.5)	.083	1.3 (1.0–1.6)	**.031**
Risk^d^				
CURB-65	1.5 (1.4–1.7)	**<.001**		
ACHU	1.4 (1.3–1.5)	**<.001**		
NEWS2	1.06 (1.02–1.10)	**.004**		
Comorbidities				
Cardiovascular disease	1.0 (.8–1.2)	.951		
Diabetes mellitus	0.9 (.7–1.2)	.502		
Chronic lung disease	1.0 (.7–1.4)	.878		

Bold indicates *P* < .05.

Abbreviations: CHBAH, Chris Hani Baragwanath Academic Hospital; HR, hazard ratio; KTHC, Klerksdorp-Tshepong Hospital Complex; PMHC, Polokwane Mankweng Hospital Complex.

^a^Defined as <12.0 g/L in women and <13 g/L in men.

^b^Defined as serum creatinine >97.2 µmol/L in women and >114.9 µmol/L in men.

^c^Defined as low (<4 mmol/L) or high (≥11.1 mmol/L).

^d^High vs low risk as defined by each classification system’s criteria.

#### Risk by Timing of Mortality

TB diagnosis (HR, 1.4; 95% CI, 1.0–1.8; *P* = .029) and low oxygen saturation on room air (HR, 1.4; 95% CI, 1.0–2.0; *P* = .036) were associated with mortality during initial admission. Males (HR, 1.7; 95% CI, 1.0–2.9; *P* = .038) had higher risk of early mortality. From 31 days postdischarge, mortality was associated with admission to Chris Hani Baragwanath Academic Hospital (HR, 1.4; 95% CI, 1.0–1.9; *P* = .027), older age (≥55 years; HR, 2.4; 95% CI, 1.2–4.9; *P* = .013), being underweight (HR, 1.5; 95% CI, 1.1–2.1), previous tobacco smoking (HR, 1.6; 95% CI, 1.2–2.2; *P* = .004), TB disease (HR, 1.6; 95% CI, 1.2–2.2; *P* = .003), anemia (HR, 1.7; 95% CI, 1.2–2.3; *P* = .001), and chronic lung disease (HR, 1.9; 95% CI, 1.2–3.0; *P* = .008). Obesity was associated with lower risk of mortality (HR, 0.5; 95% CI, .3–.8; *P* = .003).

## DISCUSSION

In this prospective cohort of adults hospitalized with CAP in South Africa, one-quarter of all those enrolled died within 1 year of follow-up, with similar mortality before and during the COVID-19 era. These deaths occurred despite high ART uptake and viral suppression in PWH and adequate pneumococcal vaccination coverage in children [8]. Half of all deaths were within 4 days of hospitalization, implying delayed presentation or rapid deterioration.

A large proportion of patients hospitalized with CAP in this study population had HIV. Bacterial pneumonia and sepsis, as important in-hospital causes of death, and the vaccine-preventable serotypes found support the well-established role of pneumococcal vaccination in adults and high-risk groups such as PWH. The median age at death of PWH was 11 years younger than PWoH, likely contributing to the longevity gap between PWH and individuals who were seronegative [5]. Although overall mortality for PWH who were virally suppressed was less than for PWH who were virally unsuppressed, mortality in participants who survived their admission approached that of PWoH almost 2 years postdischarge [9], much later than that reported by other studies in high-income countries [23]. Closing this mortality gap needs urgent attention in settings with high HIV burden such as South Africa [24]. CD4 counts taken at enrollment might have been acutely depressed, and median CD4 counts in PWH who were virally suppressed neared criteria for advanced HIV [5]. Notably, 61% of PWH who reported receiving ART were virally unsuppressed, implying insufficient adherence or resistance.

In adjusted analyses, older age (HR, 2.7 for ≥55 years and 1.8 for 45–54 years), active TB, unsuppressed HIVVL, being underweight, anemia, and low admission oxygen saturation (<94% on room air) independently predicted mortality, consistent with findings from other settings [25, 26].

COVID-19 accounted for ∼23% of underlying causes of death overall (identified in ∼50% of participants in the COVID-19 era), while HIV infection and prior TB increased risk of in-hospital mortality in these patients [26–28]. TB was the second-leading cause of death (16.9%), irrespective of HIV serostatus.

Pneumonia scoring tools categorized many participants who died as “low risk,” suggesting that severity indices should include HIV serostatus, CD4 count, and other parameters to better predict risk of death [22].

### Limitations

This observational cohort design may involve possible residual confounding. Most study recruitment and follow-up were during the COVID-19 pandemic, limiting generalizability. The large proportion of PWoH with COVID-19 pneumonia may have reduced the proportion of PWH recruited in the absence of the pandemic. We did not enroll patients who were critically ill and requiring next-of-kin consent, as access to COVID-19 wards was restricted. Importantly, COVID-19 resulted in marked reductions in influenza and RSV transmission and coinfections, which make our results less generalizable. Notably, although infant initial pneumococcal conjugate vaccine dose coverage dipped in 2020 (∼55%), completion of the primary series and booster through catch-up maintained overall final dose coverage at levels likely sufficient to sustain herd immunity (∼83%) within the same year [29]. While we excluded patients with a prior hospitalization within 30 days from enrollment, we did not record hospitalization that occurred ≥30 days to 1 year prior to the present hospitalization that may have conferred additional risk [30]. Statistical analyses in subgroups were not necessarily powered for those comparisons and were limited by missing data on HIVVL. Since study testing did not cover all plausible pathogens, outcome estimates might be confounded by other causative agents. Additionally, further investigations such as blood cultures, which were halted early due to very low positive rates, may have been of value to definitively identify cases of invasive pneumococcal disease or to better identify pneumococcal sepsis.

Documentation of a single underlying cause of death could misclassify some deaths, especially out of hospital or in those with multimorbidity [31], and lead to bias in cause-specific mortality estimates. Moreover, follow-up duration for each participant differed beyond 1 year due to COVID-related study disruptions. However, important strengths include the size of this cohort, with thoroughness of clinical assessment, laboratory confirmation of disease, and high retention in follow-up.

Preventing admission for CAP must remain a primary goal. Adult vaccination in high-risk cases against common CAP pathogens such as *S pneumoniae* should be implemented according to current guidelines, and improved earlier detection and initiation of treatment for TB could avoid progression to severe illness. Finally, strengthening of post–hospital care, including adequate prophylaxis in PWH, should be formally evaluated in clinical trials and may further reduce CAP-associated mortality [32].

## Supplementary Material

ofag594_Supplementary_Data

## Supplementary Data

Supplementary materials are available at *Open Forum Infectious Diseases* online. Consisting of data provided by the authors to benefit the reader, the posted materials are not copyedited and are the sole responsibility of the authors, so questions or comments should be addressed to the corresponding author.
