# Supplementary material for: High Mortality in Adults With Community-Acquired Pneumonia Admitted to Hospitals in South Africa Before and During the COVID-19 Pandemic, Stratified by HIV Serostatus and Viral Load: Data From a Prospective Cohort Study (2019–2021)
Source: Open Forum Infect Dis. 2026 Sep 21;13(10):ofag594. doi: 10.1093/ofid/ofag594 (PMC13660697; doi:10.1093/ofid/ofag594)
Supplement: Supplementary file 1 [file ofag594_supplementary_data.docx]

Supplementary Materials

V2.0

Potprev Mortality

**Supplementary Figure 1: Study recruitment for the HospCAP cohort of the PotPrev Study at three hospitals in South Africa before and during the COVID-19 pandemic (May 2019-October 2021) with general COVID-19 admissions at recruiting hospitals indicated (yellow line)**

^Unpublished COVID-19 admission data across study hospitals derived from hospital notifications shared with National Institutes of Communicable Diseases, 2022^
